# Circulating MicroRNA Markers for Pulmonary Hypertension in Supervised Exercise Intervention and Nightly Oxygen Intervention

**DOI:** 10.3389/fphys.2018.00955

**Published:** 2018-07-25

**Authors:** Gabriele Grunig, Christina A. Eichstaedt, Jeremias Verweyen, Nedim Durmus, Stephanie Saxer, Greta Krafsur, Kurt Stenmark, Silvia Ulrich, Ekkehard Grünig, Serhiy Pylawka

**Affiliations:** ^1^Department of Environmental Medicine and Division of Pulmonary Medicine, Department of Medicine, New York University School of Medicine, New York, NY, United States; ^2^Mirna Analytics LLC, New York, NY, United States; ^3^Thoraxklinik, Heidelberg University Hospital, Heidelberg, Germany; ^4^Clinic for Pulmonology, University Hospital Zürich, Zurich, Switzerland; ^5^Department of Medicine, University of Colorado, Aurora, CO, United States

**Keywords:** pulmonary hypertension, MicroRNA, circulating biomarker, high altitude pulmonary hypertension, supervised exercise training, nightly oxygen intervention

## Abstract

**Rationale:** Therapeutic exercise training has been shown to significantly improve pulmonary hypertension (PH), including 6-min walking distance and right heart function. Supplemental nightly oxygen also has therapeutic effects. A biomarker tool that could query critical gene networks would aid in understanding the molecular effects of the interventions.

**Methods:** Paired bio-banked serum (*n* = 31) or plasma (*n* = 21) samples from the exercise or oxygen intervention studies, respectively, and bio-banked plasma samples (*n* = 20) from high altitude induced PH in cattle were tested. MicroRNAs (miRNAs) markers were chosen for study because they regulate gene expression, control the function of specific gene networks, and are conserved across species.

**Results:** miRNAs that control muscle (miR-22-3p, miR-21-5p) or erythrocyte function (miR-451a) were chosen based on pilot experiments. Plasma samples from cattle that developed PH in high altitude had significantly higher miR-22-3p/(relative to) miR-451a values when compared to control cattle tolerant to high altitude. Measurements of miR-22-3p/miR-451a values in serum from patients receiving exercise training showed that the values were significantly decreased in 74.2% of the samples following intervention and significantly increased in the remainder (25.8%). In samples obtained after exercise intervention, a higher composite miRNA value, made of miR-22-3p and miR-21-5p/miR-451a and spike RNA, was significantly decreased in 65% of the samples and significantly increased in 35% of the samples. In the study of nightly oxygen intervention, when comparing placebo and oxygen, half of the samples showed a significant down-ward change and the other half a significant up-ward change measuring either of the miRNA markers. Samples that had a downward change in the miRNA marker following either intervention originated from patients who had a significantly higher 6-min-walking-distance at baseline (mean difference of 90 m or 80 m following exercise or oxygen intervention, respectively) when compared to samples that had an upward change in the miRNA marker.

**Conclusion:** These natural animal model and human sample studies further highlight the utility of miRNAs as future biomarkers. The different directional changes of the miRNA markers following supervised exercise training or nightly oxygen intervention could indicate different PAH molecular pathomechanisms (endotypes). Further studies are needed to test this idea.

## Introduction

Pulmonary hypertension (PH) is a progressive disease for which there is no cure. PH is characterized by increased blood pressure in the pulmonary vasculature and the right heart, and can occur as a primarily vascular disease, or associated with conditions that cause pulmonary vascular remodeling and constriction. In all instances, PH leads to adverse clinical outcomes relative to the respective disease or healthy control groups that have no PH, and can be the cause of early mortality with 2–5 years life expectancy. The current World Symposium on Pulmonary Hypertension (WSPH) classification of PH is based on a combination of patient characteristics, clinical features and cardiopulmonary hemodynamics and these WHO groups are used to inform drug treatment options (Simonneau et al., 2013). Recently, PH interventions based on supervised exercise training and nightly oxygen have been developed that have significant therapeutic effect to a degree that is expected from adding another drug for multi-drug treatment of PH (Mereles et al., 2006; Grunig et al., 2011, 2012a,b; Nagel et al., 2012; Becker-Grunig et al., 2013; Schumacher et al., 2014; Pandey et al., 2015; Ulrich et al., 2015; Ehlken et al., 2016; Gonzalez-Saiz et al., 2017; Keusch et al., 2017; Moreira-Goncalves et al., 2017; Richter et al., 2017; Leggio et al., 2018). Already after a 3-week period, supervised exercise training improved, for example, the 6MWD and hemodynamics, which are important signs for PH prognosis (Grunig et al., 2012a,b). Nightly oxygen, even after 1 week, likewise, significantly improved the 6MWD (Schumacher et al., 2014; Ulrich et al., 2015).

Aside from genetic testing and the measurement of the right-heart stress markers B-type natriuretic peptide biomarkers that would identify the molecular or cellular pathobiologic mechanism that identify the cause of PH are not established (Hoffmann et al., 2016). Further, biomarkers that could explain the effects of therapeutic interventions like supervised exercise training or nightly oxygen, would aid in the understanding of the causes of PH, and represent optimal monitoring tools for the disease. Additionally, biomarkers that could identify underlying molecular mechanisms would be important to understand the diversity of PH by characterizing subtypes of PH that are distinguished by molecular pathomechanisms [endotypes (Lotvall et al., 2011)] (Hemnes et al., 2017). This is important for personalized medicine for PH patients. The diverse molecular nature of PH is underscored by our recent understanding of mutations that can cause heritable PH. For example, recent work has identified multiple genes in multiple different molecular pathways that have gene-function altering mutations and confer greatly increased risk for developing PH. Examples include BMPR2 gene and other genes in the BMPR – transforming growth factor signaling networks, potassium channel dysfunction (KCNK3 gene), transcription factors, water channel (aquaporin gene) (Eichstaedt et al., 2017; Kimura et al., 2017; Ma and Chung, 2017; Olschewski et al., 2017; Gräf et al., 2018). Further evidence for the complex molecular cause of PH is that several risk factors have to come together to trigger disease (Viales et al., 2015; Evans et al., 2016).

MicroRNAs (miRNAs) function as epigenetic regulators of gene expression. miRNAs are small non-coding RNAs that negatively regulate gene expression by binding to the 3′UTR (untranslated region) of target messenger RNAs (mRNAs), thereby promoting mRNA degradation or suppressing the translation of the mRNA, in both cases, limiting or suppressing protein production from that specific gene. Currently, several thousands of miRNAs have been identified, each miRNA binding to several mRNAs, to control the function of several genes. In contrast to mRNA, miRNAs are actively excreted by cells and occur in the circulation (blood), miRNAs are very stable in bodily fluids, and are conserved among species. The importance of miRNA for PH has been established in recent studies (Bockmeyer et al., 2012; Brock et al., 2012; White et al., 2012). miRNAs are known for their critical function in gene-reprogramming that occurs in response to hypoxia (Hale et al., 2012), and hypoxia is an important cause for PH. Additionally, miR-204 has been identified as a critical pathogenic mediator in experimental PH, and it is down-regulated in human PH (Courboulin et al., 2011). Several miRNAs, among them miR-21 or miR-20, have been shown to target BMPR2 (Qin et al., 2009; Brock et al., 2012) and to also have critical function for the hypoxia mediated reprogramming of the pulmonary vasculature (Sarkar et al., 2010; Brock et al., 2012; Yang et al., 2012).

The current studies were designed to test the idea that circulating miRNA marker levels measured in the serum or plasma would be associated with PH in an animal model (Newman et al., 2015), and demonstrate significant change following PH-alleviating supervised exercise training (Grunig et al., 2012; Ehlken et al., 2016) or nightly oxygen (Schumacher et al., 2014; Ulrich et al., 2015) interventions in human patients.

## Materials and Methods

### Ethics Statement

All samples were from bio-repositories at the collaborating institutions. Serum and plasma samples were obtained for studies unrelated to the current experiments and then stored in respective bio-repositories at the University of Colorado (cattle plasma samples), or Universities of Zürich or Heidelberg (human samples). The previous studies (Grunig et al., 2012a; Schumacher et al., 2014; Newman et al., 2015; Ulrich et al., 2015; Ehlken et al., 2016) were performed under the supervision of the respective ethics committees at the collaborating institutions with the consent that bio-repositories can be instituted. The human participants gave written informed consent for blood sample studies for the supervised exercise intervention (Ehlken et al., 2016) and the nocturnal oxygen intervention (Ulrich et al., 2015) studies, respectively. The blood samples were obtained for other outcomes, unrelated to our study. For the current studies, the samples were sent to us from our collaborators in a de-identified manner, by persons not directly involved in the current study, such that we would never be able to access the link to the identifying data. For this reason, our research falls under the category ‘no human or animal subjects involvement’ and therefore ethics approval was not required for this research as per our Institution’s guidelines and national regulations.

### Cattle Plasma Samples

Plasma samples were analyzed from groups of cattle that were kept at high altitude (2300 m and higher) and that had received approval by the Institutional Animal Use and Care Committee at the University of Colorado (Newman et al., 2015). One group of cattle remained healthy and tolerant of the altitude with mean pulmonary artery pressures (mean PAP) of 50 mmHg and less (Newman et al., 2015). The other group had exhibited signs of intolerance to high altitude with mean PAP of 79 mmHg and more. A few cattle had an intermediate response with mean PAP of 50–79 mmHg.

### Human Plasma and Serum Samples

De-identified, plasma and serum samples from bio-repositories were received from two different centers at the Universities of Heidelberg (Grunig et al., 2012a; Ehlken et al., 2016) and Zürich (Schumacher et al., 2014; Ulrich et al., 2015), respectively. The baseline characteristics of the sample donors are listed in **Table 1**, and the diagnoses are summarized in **Table 2**. The study from the Thoraxclinic, University of Heidelberg, had the goal to test the treatment effects of a supervised exercise program that was administered for the duration of 3 weeks in PH patients (Grunig et al., 2012a; Ehlken et al., 2016). Paired serum samples had been obtained before and after the supervised exercise program for outcomes unrelated to our study. The samples were sent to us from a repository. The study from the University of Zürich had been designed to test the effects of nightly oxygen that was administered in a randomized, double blinded manner to PH patients (Schumacher et al., 2014; Ulrich et al., 2015). Each patient was randomly assigned to placebo (air) or oxygen first, and then was crossed-over to receive the other intervention. Paired plasma samples were obtained from the placebo and the oxygen parts of the study, respectively, for outcomes unrelated to our study, each taken after 1-week duration of the placebo or oxygen periods, respectively. Samples were sent to us from a repository.

**Table 1:** Characteristics of sample populations.

		U. Heidelberg	U. Zürich
		(Grunig et al., 2012a; Ehlken et al., 2016) exercise intervention	(Schumacher et al., 2014; Ulrich et al., 2015) oxygen intervention
Number of pairs		31	21
Age		50.68 ± 2.935^1^	65.14 ± 2.205
Gender	Female	21	13
	Male	10	8
NYHA class^2^	II	16	5
	III	9	16
BMI (kg/m^2^)		28.28 ± 1.239	27.33 ± 0.892
6MWD (m)		459.5 ± 23.03	438.30 ± 19.79
PH with additional diagnosis^3^		87%	100%
Mean PA pressure (mmHg)		50.54 ± 3.383	40.50 ± 3.801

*^1^Means ± SEM are indicated. ^2^Information was not available for each of the variables [e.g., NYHA (New York Heart Association) class of heart failure and mean pulmonary artery (PA) pressure]. Of the patients with missing NYHA classification (n = 6) the mean pulmonary artery pressures at rest were between 50 and 80 mmHg in three patients, and 40 mmHg in one patient. ^3^PH classifications and Additional Diagnoses are listed in **Table 2**.*

**Table 2:** Diagnoses for PH Patients.

	U. Heidelberg	U. Zürich
	(exercise intervention)	(oxygen intervention)
Total number of PH patients	31 (100%)	21 (100%)
	**Primary diagnosis**^1^	
Idiopathic PAH	19 (61%)	12 (57%)
Heritable PAH	7 (22.6%)	
Associated PAH		
connective tissue disease	2 (6.5%)	1 (4.8%)
portal hypertension	2 (6.5%)	1 (4.8%)
congenital heart disease	1 (3.2%)	
Chronic thromboembolic PH		7 (33%)
	**Additional diagnosis**^2^	
Number of patients who had additional diagnoses	27 (87%)	21 (100%)
Transient embolic episodes of the pulmonary artery	6 (19%)	
Peripheral vascular disease (hypertension, subdural hematoma, arterial blockage, chronic venous disease)	9 (29%)	8 (38%)
Chronic lung disease (COPD, airway hyperreactivity, airway obstruction, asthma)	8 (26%)	6 (28%)
Left heart disease and/or heart rhythm abnormalities	7 (23%)	6 (28%)
Anemia, or iron therapy	8 (26%)	2 (9.5%)
Auto-immune disease (e.g., insulin dependent diabetes, or autoimmune thyroiditis)	3 (9.7%)	2 (9.5%)
Kidney disease	5 (16%)	5 (24%)
Tumor	2 (6.5%)	1 (4.8%)

*^1^One primary diagnosis per patient. All patients received PH specific medication.*

*^2^One or more additional diagnoses per patient. The patients received diagnosis specific medication as needed.*

### RNA Isolation

The same volume (200 μl) plasma or serum was used for total RNA purification for all samples. Total RNA was purified using miRNAeasy Mini Kit according to the protocol of the manufacturer (Qiagen, Valencia, CA, United States) and eluted into 35 μl of water. During RNA isolation process, after the lysis and homogenization step of the manufacturer’s protocol, 1 μl of UniSp6 RNA Spike-in template (representing 10^8^ copies) was added. The Spike RNA was used as an exogenous reference for the miRNA measurements. The UniSp6 RNA Spike-in template was provided with the miRCURY LNA^TM^ Universal cDNA synthesis kit II (Exiqon, Woburn, MA, United States).

For all human plasma samples, we performed a heparinase step following RNA purification. Thirty nanograms of RNA was treated with the following mix: 0.3U of heparinase (H2519-50UN, Sigma-Aldridge, St. Louis, MO, United States) and 22U RNAse inhibitor (Invitrogen) re-suspended in 1xRT buffer from miRCURY LNA^TM^ Universal cDNA synthesis kit II, for 1 h at 25°C to remove heparin.

The cattle RNA was isolated in the same manner with addition of dialysis step prior RNA isolation, because the cattle samples were thought to contain citrate and heparin. Briefly, 200 μl of sample in dialysis tube [Slide-A-Lyser Mini Dialysis Unit, 2000 MWCO] (Thermo Fisher Scientific, Waltham, MA, United States) was dialyzed against 200 ml of 1xTE (10 mM Tris-HCl pH 8.0, 1 mM EDTA) at room temperature for 1 h. This was followed by miRNAeasy Mini Kit extraction with addition of 10^8^ copies UniSp6-Spike RNA and heparinase treatment.

Reverse transcriptase reaction was performed with miRCURY LNA^TM^ Universal cDNA synthesis kit II accordingly to manufacturer’s instructions. RNA concentrations were measured with DS-11 spectrophotometer (DeNovix, Wilmington, DE, United States).

### miRNA Expression

Real time PCR was performed in triplicate with 0.1 ng of cDNA per reaction using a 7900HT Fast Real-Time PCR instrument (Applied Biosystems/Life Technologies, Grand Island, NY, United States) in a 10 μl volume. The PCR reactions were run with LNA-modified primers and SYBR Green master mix (Exiqon, Denmark/now Qiagen) in 384-well plate under the following conditions: 95°C for 10 min, followed by 45 cycles of 95°C for 10 s and 60°C for 1 min, followed by a hold at 4°C. The following LNA-modified primers were used: hsa-miR-451a (target 5′AAACCGUUACCAUUACUGAGUU); hsa-miR-22-3p (target 5′AAGCUGCCAGUUGAAGAACUGU); hsa-miR-21-5p (target 5′UAGCUUAUCAGACUGAUGUUGA); Spike6 (target the synthetic spike RNA, UniSP6, supplied in the cDNA kit).

For the pilot study, we purchased LNA-modified primers specific for 16 additional miRNAs (hsa-let-7g-5p, hsa-miR-17-5p, hsa-miR-20a-5p, hsa-miR-20b-5p, hsa-miR-26a-5p, hsa-miR-27a-3p, hsa-miR-27b-5p, hsa-miR-30e-3p, hsa-miR-30e-5p, hsa-miR-93-5p, hsa-miR-103a-3p, hsa-miR-135a-5p, hsa-miR-142-5p, hsa-miR-150-5p, hsa-miR-204-5p, Exiqon) and performed real time PCR exactly as described above.

Raw data were then analyzed with SDS Relative Quantification Software version 2.4.1 (Applied Biosystems) to determine cycle threshold (Ct). The miRNA values that were calculated relative to synthetic spike RNA used the following equation: 1.98 to the power of [Ct of spike6 RNA – Ct of miRNA determinant], and then multiplied by 10,000 (human data) or multiplied by 10 (cattle data). The composite miRNA values were calculated as follows: 1.98 to the power of [Ct of miRNA reference(s) – Ct of miRNA determinant(s)], and then multiplied by 10,000. The miRNA values were calculated without knowledge of the characteristics of the sample donors. The full data sets that were calculated relative to synthetic spike RNA are shown in Excel data files (Supplementary Files: cattle_data.xlsx; human_large_sample_set.xlsx; human_large_mir_set.xlsx).

### Statistical Analysis

Group comparisons were performed with the two-tailed, independent Mann–Whitney *U* test, or the Wilcoxon matched pairs signed rank test as indicated. Correlations were calculated with the Spearman’s Rank Correlation test. Statistics were calculated and graphs were generated using Prism 6 (GraphPad, La Jolla, CA, United States). A *p*-value < 0.05 was considered to be statistically significant.

Hierarchical clustering was performed to calculate principal component analysis and to generate heatmaps with the freely available online R-based tool ClustVis^1^ (Metsalu and Vilo, 2015). Unsupervised hierarchical clustering was performed using Euclidean distance and complete linkage for columns (miRNA value relative to spike RNA) and rows (sample ID). The heat map graphs were re-oriented (columns and rows transposed) for best display of the data.

## Results

### Choice of miRNAs

Pilot studies were performed with RNA from whole blood and serum (*n* = 12 and *n* = 6, respectively) from paired samples from patients who had received exercise training intervention. We utilized RNA sequencing of whole blood RNA (data not shown) and literature mining to choose 18 miRNAs for a pilot study in serum samples (**Supplementary Figure S1**). Specific LNA-modified primers to hsa-let-7g-5p, hsa-miR-17-5p, hsa-miR-20a-5p, hsa-miR-20b-5p, hsa-miR-21-5p, hsa-miR-22-3p, hsa-miR-26a-5p, hsa-miR-27a-3p, hsa-miR-27b-5p, hsa-miR-30e-3p, hsa-miR-30e-5p, hsa-miR-93-5p, hsa-miR-103a-3p, hsa-miR-135a-5p, hsa-miR-142-5p, hsa-miR-150-5p, hsa-miR-204-5p, and hsa-miR-451a were detected by quantitative PCR and were calculated relative to synthetic spike RNA (**Supplementary Figure S1a**). We performed hierarchical cluster analysis to identify miRNAs that would show changed levels when comparing samples taken after and before training. We focused on the magnitude of the change, irrespectively, of the direction of the change (increased or decreased). Three miRNAs were most interesting: miR-22-3p, miR-451a, and miR-21-5p (**Supplementary Figure S1b**). We also searched for endogenous reference miRNAs that would show little, if any variations among the samples, and did not find clear candidates. However, a literature review indicated that miR-451a had been considered as a reference miRNA (Song et al., 2012), although in our samples this was not the case when miR-451a was measured against the extrinsically added spike RNA (**Supplementary Figure S1**).

### Cattle Model of High Altitude Induced PH

**FIGURE 1:**
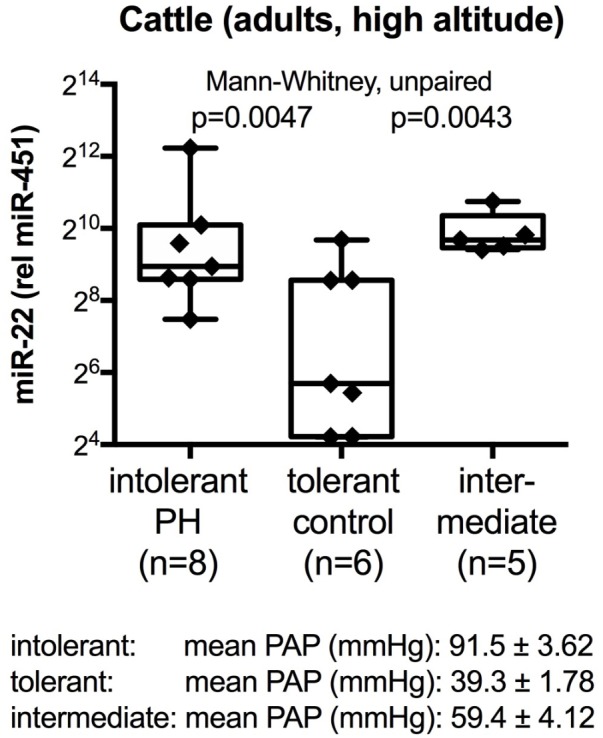
Circulating plasma miRNA values in cattle at high altitude. Box-plots with whiskers and individual points show miR-22-3p relative to miR-451a (10,000×) values in plasma samples from control cattle that were tolerant to high altitude [mean pulmonary artery pressure, mean PAP, <50 mm Hg (Newman et al., 2015)], or that developed PH (mean PAP ≥79 mmHg), or that had intermediate mean PAP. Groups were compared with the unpaired, 2-tailed Mann–Whitney test; *p* < 0.05 was considered significant.

Because the human PH condition is heterogeneous, we wanted to test the miRNAs that we had identified in the pilot studies with human samples (with focus on miR-22-3p and miR-451a) in an animal model. High altitude induced PH was chosen because this is a natural disease in cattle that has clearly identified etiology (high altitude, with the decreased oxygen tension and hypoxia challenge) with genetic predisposition in the EPAS-1 (Endothelial PAS Domain-Containing Protein 1) gene, also known as hypoxia inducible factor 2-alpha. Additional reasons to choose the cattle model included the possibility to measure pulmonary arterial pressures (PAP) by catheterization, even in the clinical setting, and the cross-species conservation of the miRNAs between cattle and human. Plasma samples from cattle that were tolerant to high altitude (mean PAP less than 50 mmHg), cattle that had developed PH (mean PAP more than 79 mmHg) and cattle with intermediate mean PAP values were analyzed. The data showed significantly decreased miR-22-3p determined relative to miR-451a values in the plasma samples from the tolerant (control) cattle, as compared to the intolerant and intermediate groups (**Figure 1**). miR-22-3p and miR-451a are of molecular interest in PH, particularly in PH associated with oxygen consumption, because miR-22-3p has been shown to regulate muscle function, including skeletal (Eisenberg et al., 2007; Schweisgut et al., 2017), heart and smooth muscle (Huang et al., 2013; Huang and Wang, 2014; Zhao et al., 2015), control estrogen signaling by targeting the estrogen receptor (Pandey and Picard, 2009), while miR-451a controls erythrocyte function (Dore et al., 2008; Yu et al., 2010) and skeletal muscle function (Davidsen et al., 2011).

### Choice of Reference miRNAs for the Human Test

Having confirmed the utility of miR-22-3p and miR-451a as potential biomarkers for PH in the cattle model, we wanted to further understand the relationships between the miRNA measurements, including also miR-21-5p. Hierarchical cluster analysis was performed on all data from both intervention studies (exercise or oxygen, respectively, with 52 donors and 104 samples in total). The results are presented in a heatmap display (**Supplementary Figure S2**). The cluster analysis showed that miR-21-5p and miR-22-3p clustered together, separately from miR-451a, Additionally, the clustered miRNA’s separated the before and after intervention measurements in 46 donors, and only 6 donor data clustered together (**Supplementary Figure S2**).

For miR-22-3p relative to miR-451a and additionally for each of the studied miRNA’s we calculated the miRNA level values relative to spike RNA and determined the fold change after training or oxygen, respectively. We then determined correlations of the fold-change values (**Table 3**). The calculations showed that the fold change determined in miR-22-3p (relative to spike) was significantly correlated with the fold change in miR-21-5p (relative to spike) in both the training and the oxygen intervention studies, respectively (**Table 3**). This prompted us to also compare the fold-change in composite miRNA values, combining miR-22-3p and miR-21-5p, as a way to diminish the significance of technical variations (minute pipetting errors for example) as causes for variations in the qPCR quantification values. As for the reference miRNA composite choice, we used miR-451a plus spike RNA, because spike RNA is the invariant component of our test, as the same number of copies (10^8^) of spike RNA were added to each 200 μl of sample used for the RNA isolation. The composite miRNA marker was then calculated as miR-22-3p + miR-21-5p relative to miR-451a + spike RNA. The composite marker (miR-22-3p + miR-21-5p relative to miR-451a + spike RNA) was significantly correlated with the values for miR-22-3p relative to miR-451a in both the exercise training and oxygen intervention studies, respectively (**Table 3**).

**Table 3:** Correlations between different types of miRNA measurement readouts.

(A) miRNA marker change following exercise training intervention (*n* = 31 pairs).						
*X* – Variable	miR-22/miR451	miR-22/miR451	miR-22/miR451	miR-22/spike	miR-451/spike	miR-22/miR451
*Y* – Variable	miR-22/spike	miR-451/spike	miR-21/spike	miR-21/spike	miR-21/spike	miR-22 + miR-21/miR- 451 + spike
Spearman’s *R*	0.3156	-0.348	0.09699	0.704	0.629	0.5986
*P*-Value	0.0838	0.055	0.6037	**<0.0001**	**0.0002**	**0.0004**
(B) miRNA marker change following nightly oxygen intervention (*n* = 21 pairs).						
*X* – Variable	miR-22/miR451	miR-22/miR451	miR-22/miR451	miR-22/spike	miR-451/spike	miR-22/miR451
*Y* – Variable	miR-22/spike	miR-451/spike	miR-21/spike	miR-21/spike	miR-21/spike	miR-22 + miR-21/miR-451 + spike
Spearman’s *R*	0.7636	-0.2558	0.3143	0.5221	0.2494	0.8688
*P*-Value	**<0.0001**	0.2630	0.1653	**0.0152**	0.2757	**<0.0001**

*Correlations and significance were calculated with Spearman’s rank correlation test on the fold-change after training relative to baseline, or oxygen relative to placebo, respectively. A p < 0.05 was considered significant (bold).*

### miRNA Value Changes After Supervised Exercise Training or Oxygen Interventions

**FIGURE 2:**
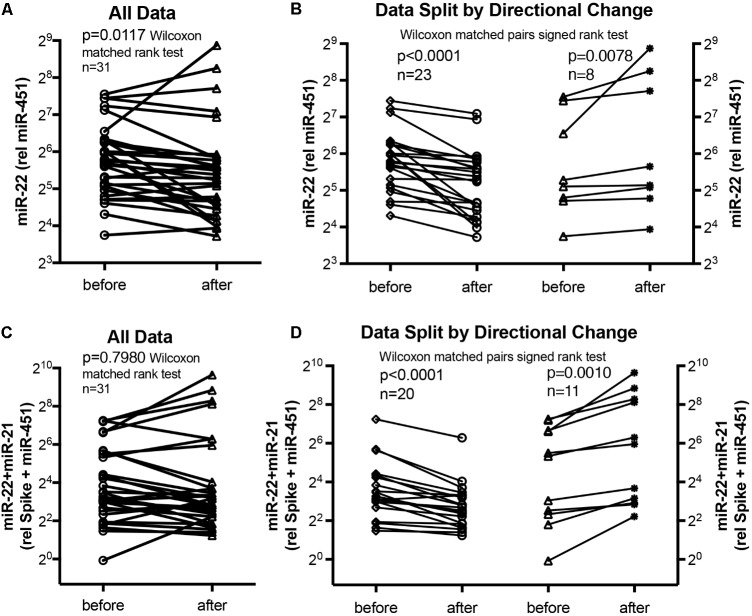
Circulating serum miRNA values in the supervised exercise training intervention study. Symbols and lines graphs show miR-22-3p relative to miR-451a (10,000×) values **(A,B)**, or miR-22-3p + miR-21-5p relative to spike-RNA + miR-451a (10,000×) values **(C,D)** in serum samples obtained from patients (Ehlken et al., 2016; Grunig et al., 2012a) before and after exercise training intervention. All of the data are plotted in **(A,C)**; in **(B,D)** the data are separated by the directional change in the samples after exercise training intervention. Groups before and after intervention were compared with the Wilcoxon matched pairs signed rank test; *p* < 0.05 was considered significant.

**FIGURE 3:**
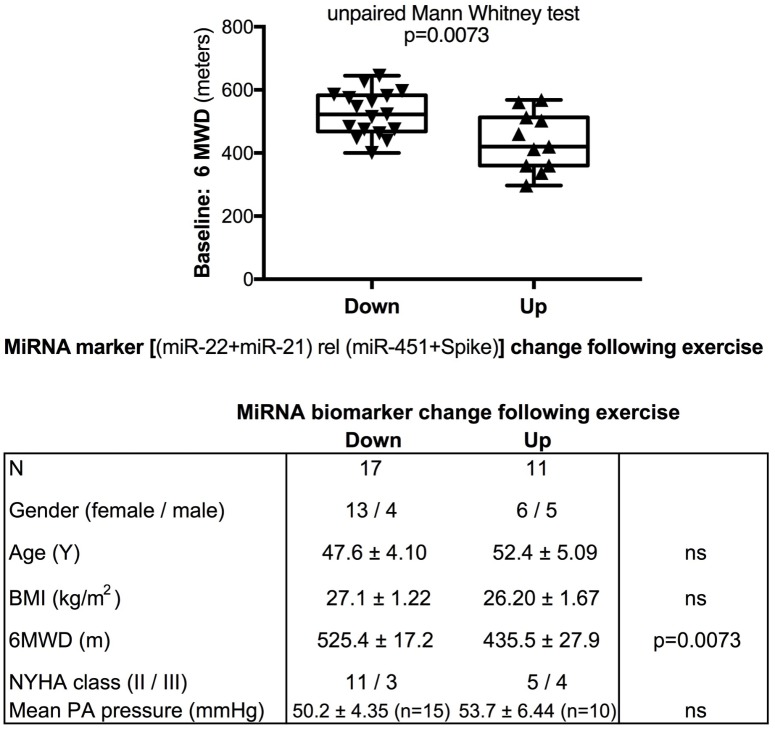
Baseline 6-min walking distance in the exercise training intervention study - comparison with directional change of the miRNA marker. Box-plots with whiskers and individual points show 6-min-walking-distance (6MWD, m) measured at baseline examination from patients in the exercise training intervention study. The data were grouped by the directional change of the miR-22-3p + miR-21-5p relative to spike-RNA + miR-451a (10,000×) values after versus before exercise training intervention: down or up, as shown in **Figure 2D**. Groups were compared with the unpaired, 2-tailed Mann–Whitney test; *p* < 0.05 was considered significant, ns is not significant. The table graph lists the characteristics of the sample donors for each group. For this analysis, samples from three patients who had a body mass index (BMI) greater that 40 kg/m^2^ and who achieved 6-min-walking-distances between 150 and 190 m were omitted; two of these three patients were NYHA class III, one was NYHA class not determined.

**FIGURE 4:**
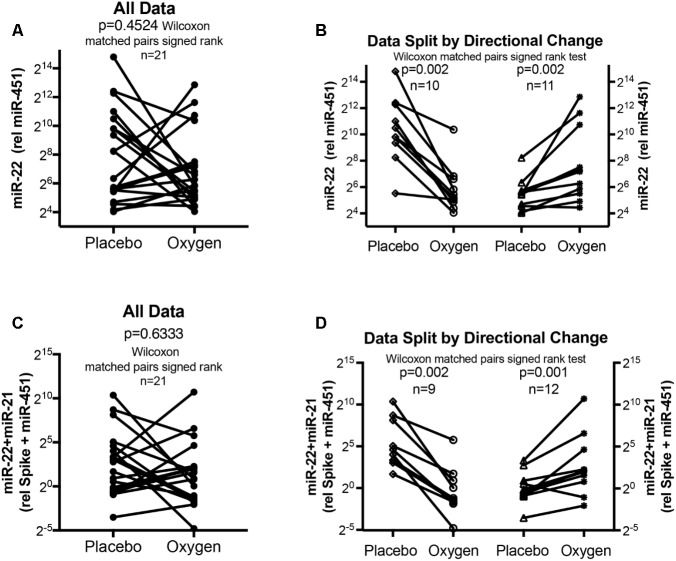
Circulating serum miRNA values in the oxygen intervention study. Symbols and lines graphs show miR-22-3p relative to miR-451a (10,000×) values **(A,B)**, or miR-22-3p + miR-21-5p relative to spike-RNA + miR-451a (10,000×) values **(C,D)** in plasma samples obtained from patients (Schumacher et al., 2014; Ulrich et al., 2015) given placebo (air) or oxygen in a cross-over design. All of the data are plotted in **(A,C)**; in **(B,D)** the data are separated by the directional change in the samples after oxygen intervention. Groups before and after intervention were compared with the Wilcoxon matched pairs signed rank test; *p* < 0.05 was considered significant.

**FIGURE 5:**
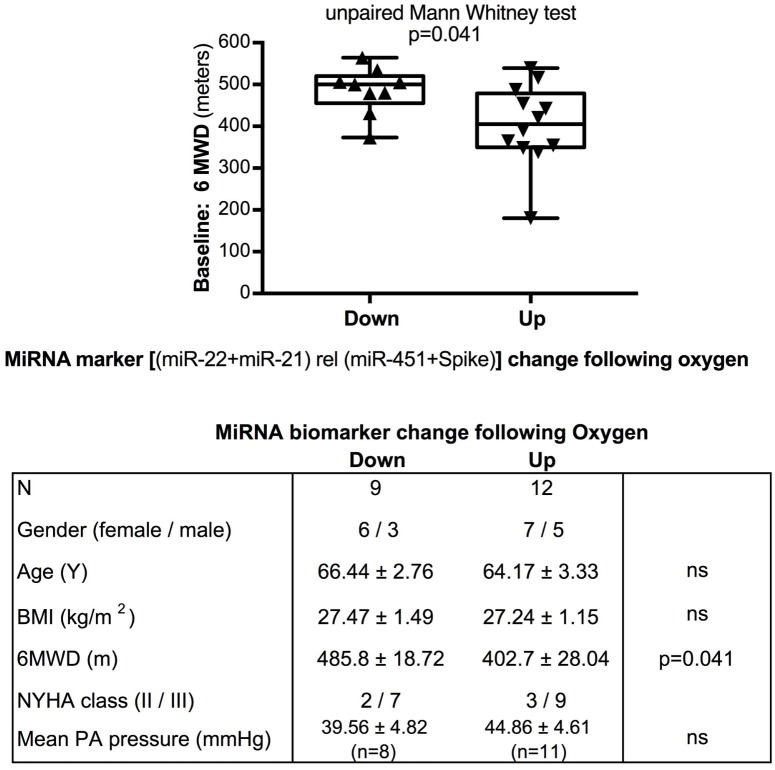
Baseline 6-min walking distance in the oxygen intervention study – comparison with directional change of the miRNA marker. Box-plots with whiskers and individual points show 6-min-walking-distance (6MWD, m) measured at baseline examination from patients in the oxygen intervention study. The data were grouped by the directional change of the miR-22-3p + miR-21-5p relative to spike-RNA + miR-451a (10,000×) values after oxygen versus placebo: down or up, as shown in **Figure 4D**. Groups were compared with the unpaired, 2-tailed Mann–Whitney test; *p* < 0.05 was considered significant, ns is not significant. The table graph lists the characteristics of the sample donors for each group.

We used miR-22-3p relative to miR-451a and miR-22-3p + miR-21-5p relative to miR-451a + spike RNA values to compare changes following supervised exercise training (**Figures 2**, **3**) or nightly oxygen (**Figures 4**, **5**), respectively. In the exercise intervention, the serum levels of miR-22-3p relative to miR-451a were significantly decreased (*p* = 0.012) following training, however, the direction of change was clearly increased in some of the samples (**Figure 2A**). The divergence of the direction of the change in miRNA values could be clearly shown by dividing the samples based on the direction of the fold change (**Figure 2B**). The divergence of the direction of change in miRNA values was even more pronounced when miR-22-3p + miR-21-5p relative to miR-451a + spike RNA was calculated (**Figures 2C,D**). Comparing miRNA directional changes to baseline disease characteristics we found a significant difference in the 6-min-walking distance (**Figure 3**). Samples that showed a decreased directional change in the miRNA marker [miR-22-3p + miR-21-5p relative to miR-451a + spike RNA] (**Figure 2D**) were obtained from patients who had a significantly longer 6-min-walking distance at baseline (**Figure 3**). For this analysis, we omitted samples from 3 patients who had a BMI greater that 40 kg/m^2^ and who achieved 6-min-walking-distances between 150 and 190 m.

**FIGURE 6:**
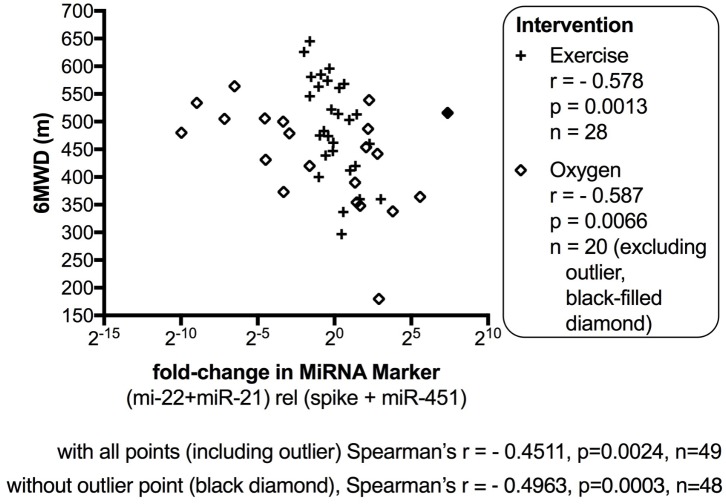
Correlation between change in miRNA marker and 6-min-walking-distance, supervised exercise training and nightly oxygen intervention data combined. The fold-change in miR-22-3p + miR-21-5p relative to spike-RNA + miR-451a (10,000×) values (after/before exercise, or oxygen/placebo, respectively) were plotted against 6-min walking distance (m) measured at baseline examination from the patients. The data from the exercise training intervention study were plotted with × symbols, and the data from the nightly oxygen intervention study were plotted as clear/black-filled diamond symbols. The black-filled diamond represents one outlier data point, and correlations were calculated with and without the outlier point as indicated. The correlation was calculated with Spearman’s rank correlation test; *p* < 0.05 was considered significant. For this analysis, samples from three patients who had a body mass index (BMI) greater that 40 kg/m^2^ and who achieved 6-min-walking-distances between 150 and 190 m were omitted.

The data from the nightly oxygen intervention study showed that we measured higher miRNA marker values as compared to the values measured in the exercise intervention study (**Figure 4** compared to **Figure 2**). This is likely due to the different materials being studied: serum in the exercise intervention study (**Figure 2**), plasma in the oxygen intervention study (**Figure 4**). This idea is supported by the data from cattle plasma samples that show miRNA marker values in a similar range as the human plasma samples (**Figure 1** compared to **Figure 4**). As observed in the exercise intervention study (**Figure 2**), the oxygen intervention study showed that significant changes in the direction of the miRNA values followed individual variation, with approximately half demonstrating decreased, and the other half increased miRNA marker values (**Figure 4**). This was found both with miRNA marker values being calculate by miR-22-3p relative to miR-451a, or miR-22-3p + miR-21-5p relative to miR-451a + spike RNA (**Figures 4B,D**). To understand if the fold change difference could represent different PH endotypes, we compared the groups of patients whose miRNA value was either significantly decreased or increased following oxygen intervention. We found a significant difference between these groups in the 6MWD recorded at baseline (**Figure 5**). As we had already seen in the data from the exercise intervention study (**Figure 3**), the group that had an upward change in the miRNA marker following oxygen intervention had a significantly lower 6-min-walking-distance at baseline (**Figure 5**). Further, in both intervention studies, there was a higher percentage of males in the group with an upward direction of the miRNA marker change, but the difference to the group that had a down-ward change was not significant (**Figures 3**, **5**). To further confirm the possibility that the 6-min-walking-distance at baseline could predict, or be correlated, with the directional, fold-change of the miRNA marker following intervention, we plotted fold-change of serum or plasma miR-22-3p + miR-21-5p relative to miR-451a + spike RNA (**Figure 6**). The data from the exercise training and the nightly oxygen intervention studies were combined to increase the number of observations for analysis and to query for generalizable impact (**Figure 6**). For this analysis, we omitted samples from three patients who had a BMI greater that 40 kg/m^2^ and who achieved 6-min-walking-distances between 150 and 190 m. The analysis showed a significant correlation calculated with Spearman’s rank correlation test between 6MWD at baseline and the fold change of the miRNA marker (miR-22-3p + miR-21-5p relative to miR-451a + spike RNA, **Figure 6**).

## Discussion

Our studies identified circulating miRNA markers composed of miR-21, miR-22, and miR-451 that showed significant change in a natural animal model (Newman et al., 2015), and in two intervention studies [supervised exercise training (Grunig et al., 2012a; Ehlken et al., 2016) and nightly oxygen (Schumacher et al., 2014; Ulrich et al., 2015)] in human PH patients. In the human studies, the change in miRNA value following intervention was significantly correlated with the 6MWD at baseline. While the present study did not evaluate the genes (mRNA) controlled by the miRNAs, a literature search shows that, for example, miR-21 directly targets BMPR2 (Qin et al., 2009). Both miR-21 and miR-22 control fibrosis in the lungs (Liu et al., 2010), or kidney, respectively (Long et al., 2013). Further, miR-21 and miR-22 are dysregulated in some primary muscle diseases (Eisenberg et al., 2007) and miR-451 is differentially regulated in low and high responders of a specific muscle training program of healthy volunteers (Davidsen et al., 2011). miR-22 is also a critical regulator of cardiac muscle health, and smooth muscle differentiation (Huang et al., 2013; Huang and Wang, 2014; Zhao et al., 2015). miR-451a is a critical regulator of erythropoiesis and erythrocyte function (Dore et al., 2008; Yu et al., 2010).

Our data add to the knowledge of miRNA function in PH (Bockmeyer et al., 2012; White et al., 2012; Deng et al., 2015; Caruso et al., 2017) that has thus far mainly focused on miRNA control of endothelial cells (Huertas et al., 2018), vascular smooth muscle cells, the right heart, the important PH risk factor gene, BMPR2, and the PH inducing hypoxia miRNAs (hypoxamirs), with particular significance of miR-204 (Courboulin et al., 2011), miR-20 (Brock et al., 2012), and miR-21 (Qin et al., 2009; Sarkar et al., 2010; Yang et al., 2012). miRNAs have been further shown to have critical significance for the dysfunction of endothelial cell metabolism and function (miR-124) in PH and mitochondrial dysfunction of fission and calcium transport causing hyper-proliferation of smooth muscle cells (miR-34a-3p, miR-138, miR-25) in PH (Caruso et al., 2017; Hong et al., 2017; Chen et al., 2018).

Our studies highlight the significance of skeletal muscle function in PH as miR-21, miR-22, and miR-451a share skeletal muscle as one of their cellular targets (Eisenberg et al., 2007; Davidsen et al., 2011; Schweisgut et al., 2017). Furthermore, miR-451 is known for its control of erythrocyte function (Dore et al., 2008; Yu et al., 2010). This may be the reason why the composite miRNA marker value that contained miR-22, miR-21, miR-451a, and spike-RNA was correlated with the 6-min-walking distance measured at baseline by the patients who participated in the exercise training intervention or oxygen intervention studies, respectively. Skeletal muscle function and optimal erythrocyte transport of oxygen are critical for achieving optimal walking distances, and muscle wasting has been described in PH patients (Marra et al., 2015). Optimized erythrocyte function is an important adaptation to high altitude hypoxia (Song et al., 2017). It is intriguing that miR-451a has been described as one of the miRNAs that demonstrate differential response directions in healthy young men who underwent resistance exercise training and had varying degrees of muscle mass gain (Davidsen et al., 2011). Therefore, future studies have to be designed to distinguish between the possibilities that the divergent directional changes in miRNA marker values that we observed, are already an intrinsic personal trait due to skeletal muscle responsiveness prior to the development of PH, or are developed as consequence of the PH disease.

Our studies provide an example for the importance of using animal models for biomarker discovery in PH because of the variations intrinsic to human disease. In our case, there was no optimal experimental mouse or rat model of exercise or oxygen intervention in PH available. The cattle model has the advantages of being a natural disease, and, importantly, control as well as PH cattle are perfectly matched (Newman et al., 2015). Control animals share the same environment as the animals that develop to PH including high altitude, food, pasture, animal housing (Newman et al., 2015). In contrast, in experimental models of hypoxia induced PH, the experimental group is exposed to hypoxia, the control group is not. In our case where the goal was to identify miRNA markers that track PH, it was essential that the control animal group was also exposed to hypoxia, as hypoxia itself induces changes in the expression of a large set of miRNA markers, called hypoxamirs (Hale et al., 2012). Furthermore, our data in cattle may be hypothesis generating to further develop the understanding of the regulation of PAP at high altitude in humans by miRNAs (Blissenbach et al., 2018).

Our study in samples from human patients was designed with the heterogeneity of PH in focus, although all patients responded favorably to the supervised exercise training intervention (Grunig et al., 2012a; Ehlken et al., 2016), or nightly oxygen (Schumacher et al., 2014; Ulrich et al., 2015) intervention, respectively. The heterogeneity of PH is highlighted by the heterogeneity of gene mutations in heritable PH (Eichstaedt et al., 2017; Ma and Chung, 2017; Gräf et al., 2018). The heterogeneity of the underlying molecular mechanisms that cause disease has prompted the development of tools for personalized medicine (Hemnes et al., 2017). Big data, OMICS studies are currently conducted to fine-map clinical, physiological, imaging and molecular parameters that are then used to better define clinical subtypes and to identify disease endotypes (Hemnes et al., 2017). One example in PH is the pulmonary vascular disease OMICS (PVDOMICS) study (Hemnes et al., 2017). Our study, in contrast, did not have the power based on thousands of individual data points to draw this fine-map of PH. Instead we used three different sample cohorts to identify miRNA markers that would demonstrate significant changes in the biomarker: a high altitude animal model of PH (Newman et al., 2015), and PH interventions in humans consisting either of supervised exercise training (Grunig et al., 2012a; Ehlken et al., 2016) or nightly oxygen (Schumacher et al., 2014; Ulrich et al., 2015). The significant difference in the homogenous animal model, in contrast to the individual differences in the direction of the change of the miRNA marker together with differences among groups with respect to baseline 6-min-walking-distance in human PH, implies that the biomarker may have distinguished PH endotypes. However, further studies using large patient cohorts are needed to confirm this idea.

## Conclusion

Our analysis identified circulating miRNAs that control muscle and erythrocyte function (miR-22-3p, miR-21-5p, miR-451a) that may have utility as biomarkers of PH progression and responses to intervention. The strength of our data is in the consistency across different patient groups given supervised exercise training or oxygen intervention, respectively, consistency across sample processes (serum versus plasma), and confirmation in a natural animal model. Further research in larger patient groups and in additional data sets is needed to validate our data and to test the idea that different PH endotypes are, in part, the cause for the individual variation in the direction of the miRNA marker changes in response to the training or oxygen interventions. Future studies will also need to address the relationship between the miRNA markers and commonly used markers in PH (e.g., B-type natriuretic peptide). Furthermore, future studies will need to be designed to identify the molecular mechanisms by which exercise training or oxygen interventions provide feed-back signals via the changed levels of circulating miRNAs to the pulmonary vasculature, and the clinical PH phenotype, because miR-21 and miR-22 also have functions in the pulmonary vasculature (Sarkar et al., 2010; Yang et al., 2012; Zhao et al., 2015) and in the heart (Huang et al., 2013; Huang and Wang, 2014).

## Author Contributions

GG and SP conceived the study, performed the experiments, and wrote and edited the manuscript. CE, JV, SS, GK, KS, SU, and EG established and maintained the sample bio-repositories. JV and SS sent samples to GG and SP for study. CE, ND, SU, and EG edited the figures and manuscript.

## Conflict of Interest Statement

GG and SP are the co-founders of Mirna Analytics. The remaining authors declare that the research was conducted in the absence of any commercial or financial relationships that could be construed as a potential conflict of interest.

## Footnotes

1 https://biit.cs.ut.ee/clustvis/

## Supplementary Material

FIGURE S1 Hierarchical clustering of serum miRNA levels determined relative to synthetic spike RNA from samples obtained before (B) or after (A) exercise intervention. **(a)** Full pilot study miRNA panel. Note that miR-22-3p and miR-21-5p are in clusters distinct from miR-451a. Also note that samples from a single donor are listed together (box) but not clustered together. **(b)** Selected miRNAs. Note that miR-22-3p and miR-21-5p are clustered together apart from miR-451a. Note that none of the paired samples are listed together.

FIGURE S2 Hierarchical clustering of serum or plasma miRNA levels determined relative to synthetic spike RNA from *n* = 52 paired samples, total *n* = 104. The samples were obtained before (B) or after (A) exercise intervention at the University of Heidelberg (HD), or following placebo (P) or oxygen (O) intervention at the University of Zurich (ZH), respectively. Samples from 46 donors clustered apart; the samples from 6 donors were either listed or clustered together and are indicated (boxes).

## Abbreviations

6MWD: 6-min walking distance
BMI: body mass index
BMPR2: bone morphogenetic protein receptor 2
miRNA: MicroRNA
PAP: pulmonary artery pressure
PH: pulmonary hypertension
