# Supplementary figures and images for: Circulating MicroRNA Markers for Pulmonary Hypertension in Supervised Exercise Intervention and Nightly Oxygen Intervention
Source: Front Physiol. 2018 Jul 25;9:955. doi: 10.3389/fphys.2018.00955 (PMC6068281; doi:10.3389/fphys.2018.00955)

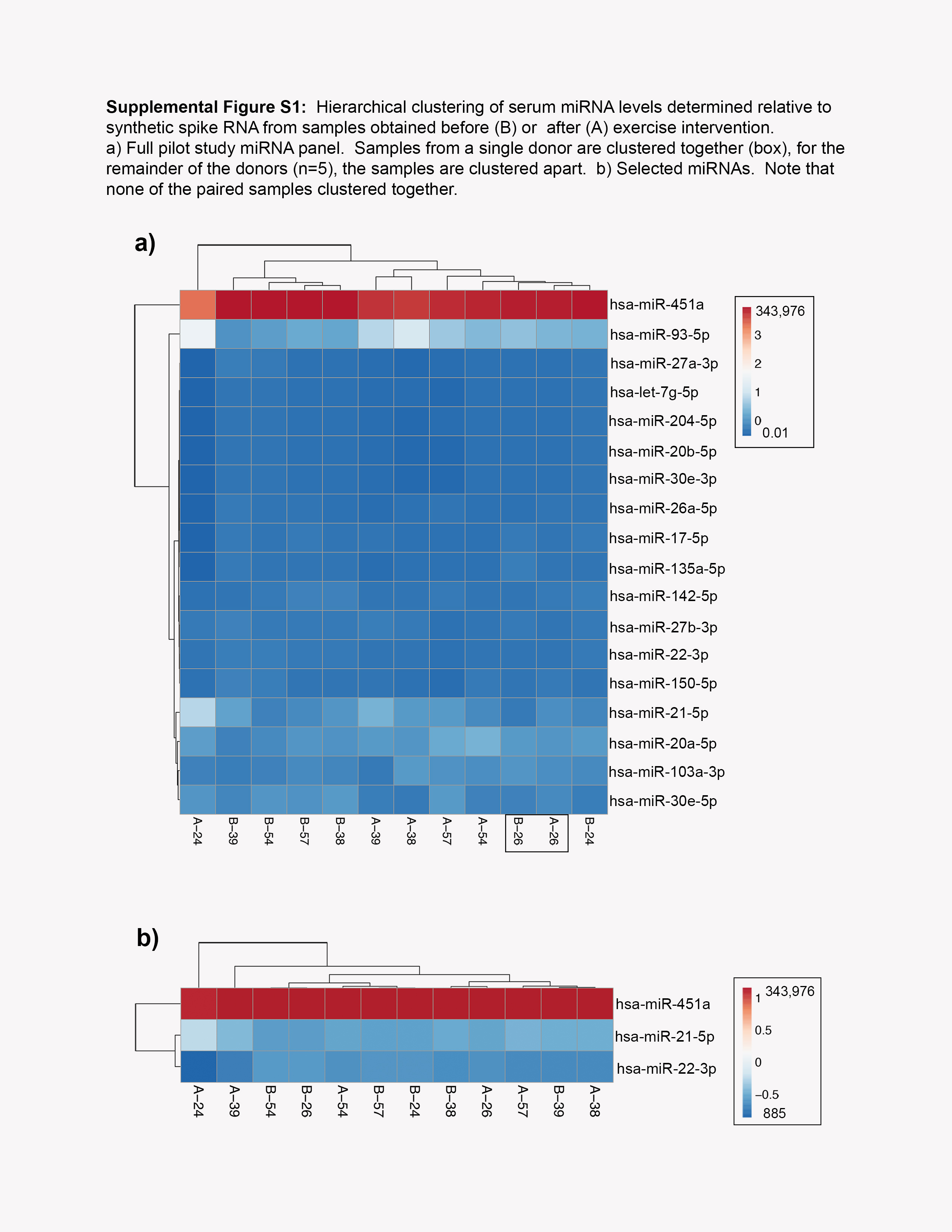

Supplement: FIGURE S1 — Hierarchical clustering of serum miRNA levels determined relative to synthetic spike RNA from samples obtained before (B) or after (A) exercise intervention. (a) Full pilot study miRNA panel. Note that miR-22-3p and miR-21-5p are in clusters distinct from miR-451a. Also note that samples from a single donor are listed together (box) but not clustered together. (b) Selected miRNAs. Note that miR-22-3p and miR-21-5p are clustered together apart from miR-451a. Note that none of the paired samples are listed together. [file Image_1.JPEG]

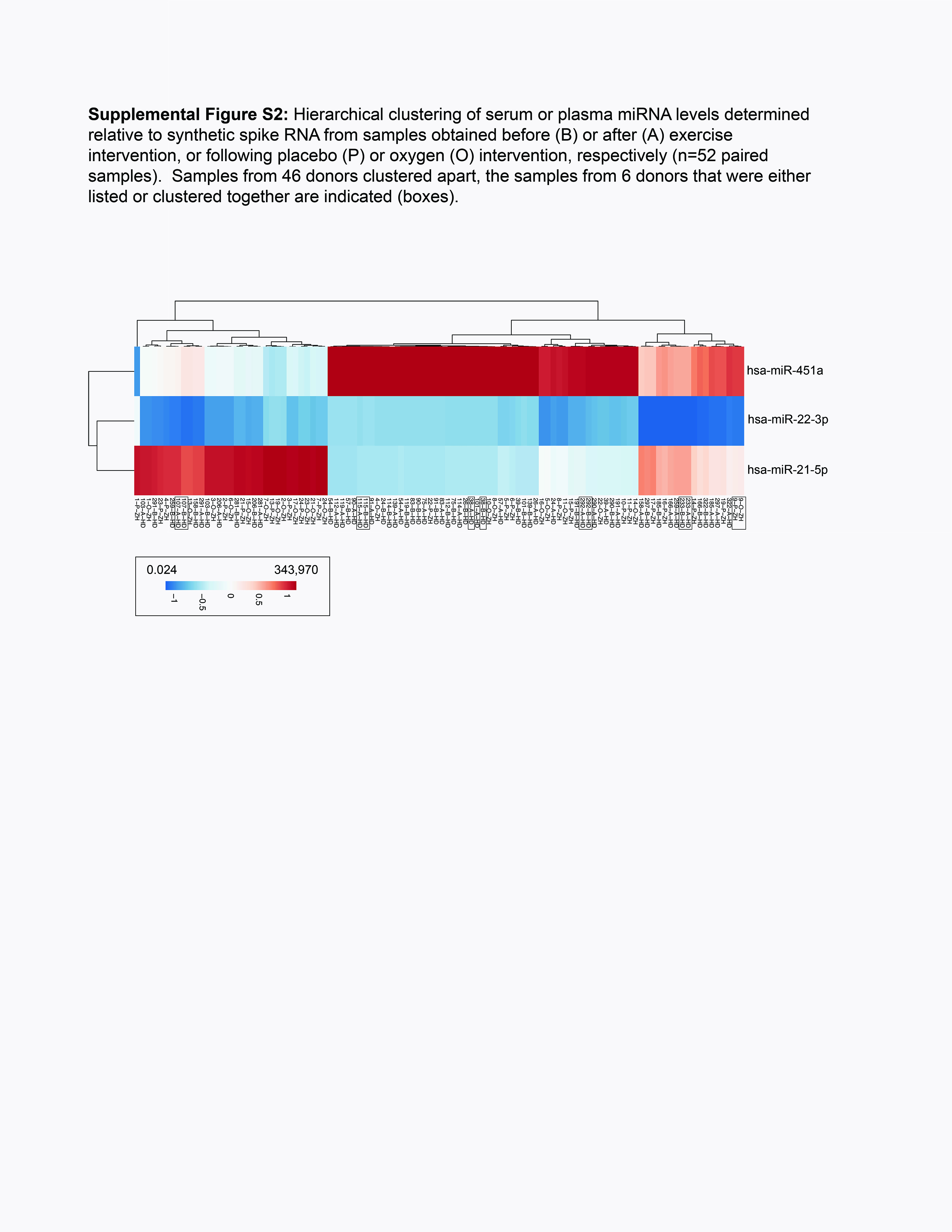

Supplement: FIGURE S2 — Hierarchical clustering of serum or plasma miRNA levels determined relative to synthetic spike RNA from n = 52 paired samples, total n = 104. The samples were obtained before (B) or after (A) exercise intervention at the University of Heidelberg (HD), or following placebo (P) or oxygen (O) intervention at the University of Zurich (ZH), respectively. Samples from 46 donors clustered apart; the samples from 6 donors were either listed or clustered together and are indicated (boxes). [file Image_2.JPEG]
